# Enhanced LDM for Next-Generation Digital Broadcasting Transmission

**DOI:** 10.3390/s21051716

**Published:** 2021-03-02

**Authors:** Xianzheng Deng, Xin Bian, Mingqi Li

**Affiliations:** 1 Shanghai Advanced Research Institute, Chinese Academy of Sciences, Shanghai 201210, China; dengxianzheng@sari.ac.cn (X.D.); bianx@sari.ac.cn (X.B.); 2 University of Chinese Academy of Sciences, Beijing 100049, China

**Keywords:** LDM, multi-service transmission, LDPC, FDM, broadcasting

## Abstract

In the traditional layered division multiplexing (LDM) system, by simply adjusting the injection level, the reception performance of the core layer (CL) mobile services will be decreased significantly, resulting in the deterioration of system coverage performance. Thus, it is necessary to improve the performance of the enhanced layer (EL) service reception without affecting the reception threshold of CL service. To achieve this, in this paper, an enhanced LDM (En-LDM) scheme that supports multi-service transmission is proposed for the next-generation broadcasting network. In this scheme, at the transmitter end, part of the low-density parity-check (LDPC) coded data stream of fixed service conveyed in the EL will be extracted out through puncturing, and then, it will be transmitted over the CL of the LDM signal along with the original CL data in a frequency domain multiplexing (FDM) manner. At the receiving end, the punctured data of the EL fixed service will be recovered with a higher signal-to-noise ratio (SNR). Compared to the traditional LDM scheme, the proposed En-LDM scheme can significantly improve the reception performance of fixed services without decreasing the SNR threshold of mobile services. Moreover, the En-LDM can achieve a higher channel capacity than that of the traditional LDM for both the fixed services and the overall services. The superiority of the proposed En-LDM scheme over the traditional LDM scheme is validated by the simulation under the additive white Gaussian noise (AWGN) and fading channels.

## 1. Introduction

In recent years, as the types and quantities of handheld portable devices and fixed terrestrial broadcast receiving devices have developed in full swing, people’s needs for multimedia content are becoming increasingly diverse. In order to meet the growing demand for multi-service transmission and to make more efficient use of spectrum resources in future broadcasting networks, several next-generation digital TV broadcasting standards have been announced successively around the world. For example, in June 2008, the European second-generation digital terrestrial TV broadcast transmission standard DVB-T2 was announced [1]. In July 2015, China’s next-generation digital TV broadcasting system digital terrestrial television multimedia broadcasting-advanced (DTMB-A) was adopted by the International Telecommunication Union (ITU) [2]. In June 2017, the physical layer protocol (A/322) of the Advanced Television Systems Committee 3.0 (ATSC 3.0) was approved as the final standard [3,4,5,6].

Compared to existing standards, ATSC 3.0 standard can provide better flexibility, more robustness, and higher spectrum efficiency [7,8,9,10,11,12,13]. A key physical layer technique in ATSC 3.0 is the so-called layer division multiplexing (LDM). LDM is a non-orthogonal multiple access (NOMA) technology. Both NOMA and point-to-multipoint (P2MP) have been identified as essential technologies for 5G to make more efficient use of the spectrum [14,15,16]. Ref. [17] discussed the application of LDM as an enabling technology for 5G and proved that the P2MP subsystem with LDM can deliver high-quality broadcast services using the broadband infrastructure. The basic principle of LDM is that the second layer of the data stream is superimposed to the original data layer to form a composite signal. Usually, the second layer of the data stream is called the enhanced layer (EL, also called the upper layer) signal, and the original data layer is the core layer (CL, also called the lower layer) signal. All the two signals share the same bandwidth except that the power of the CL signal is higher than that of the EL signal. The power difference between CL and EL signals is characterized by the power injection level. Compared with traditional time division multiplexing (TDM) and frequency division multiplexing (FDM) technologies, the LDM technology can provide a lower received signal-to-noise ratio (SNR) threshold and higher spectral efficiency [18,19,20]. Consider a typical application scenario of LDM where there are two differentiated services, namely mobile services and fixed services. The former is used for outdoor and portable reception of high-definition signals, which can usually tolerate lower data rates and is carried by the more robust CL; the latter is exploited to transmit ultra-high-definition television signals, which usually require higher data rates and are carried by the lower power EL [21,22,23,24,25].

In terms of coverage and networking, note that there is a phenomenon that receiving methods in different scenarios have different gain differences. In order to achieve the same coverage for different services in the LDM system, the requirements for the receiver antenna height correction and gain correction value will be more stringent. Therefore, in actual reception, the receiver antenna form and installation will directly affect the service effective coverage. For example, for the high bit rate services transmitted by EL, when the antenna gain and installation height cannot reach the value of the service plan, a large range of EL holes may appear within the effective coverage of CL, which is more obvious in urban environments [23,26]. Although the coverage of the EL signal can be enhanced by simply adjusting the injection level, it will significantly affect the reception performance of CL mobile services. Therefore, it is necessary to improve the performance of EL service reception without affecting the reception threshold of CL service.

To achieve that, this paper proposes an enhanced LDM (En-LDM) solution that supports the multi-service transmission, which enables two service streams with different SNR ranges to be simultaneously transmitted in the same frequency band, while the SNR threshold for the fixed service is higher than that for mobile services. In the proposed scheme, part of the data extracted from the original EL fixed service through puncturing is transmitted through the CL of the LDM. The SNR of this part of the fixed service data is higher than that of the remaining fixed service data of the EL, which is more beneficial for decoding correctly. At the receiving end, after the data retrieved by the puncturing is restored in accordance with the original puncturing rules, the overall demodulation performance of the EL fixed service is improved, and the transmission rate of the fixed service is also increased accordingly. Among them, the selection of the puncturing method is based on the low-density parity-check-encoded (LDPC-encoded) bit puncturing method, which is more flexible, so that the modulation constellation of the fixed service data puncturing part can be selected according to the actual channel conditions. The technical comparison among the proposed En-LDM scheme and several typical LDM schemes is illustrated in Table 1.

The rest of this paper is organized as follows. The traditional LDM scheme is briefly introduced in Section 2. The En-LDM scheme mechanism will be proposed in Section 3, along with the theoretical analysis and numerical simulation on the channel capacity of each service. In Section 4, the system performance of the En-LDM scheme will be presented and evaluated. Finally, conclusions are drawn in Section 5.

## 2. Overview of the Traditional LDM Scheme

**Figure 1:**
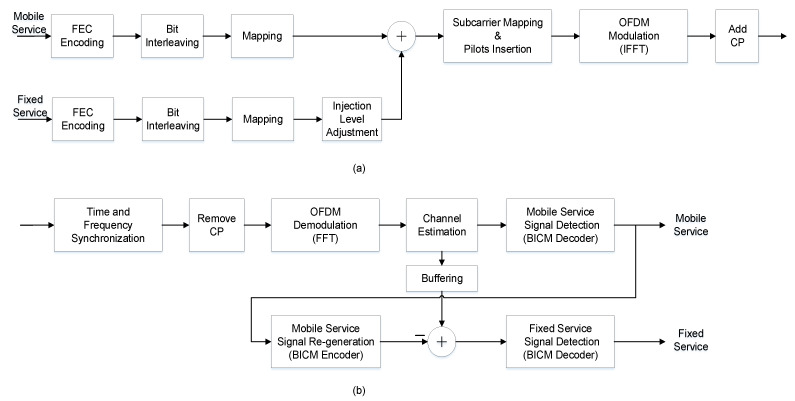
Block diagram of the traditional LDM system transceiver: (**a**) two-layer LDM transmitter; (**b**) two-layer LDM receiver.

The basic structure diagram of the transmitter and receiver of the two-layer LDM in the ATSC 3.0 system is shown in Figure 1.

At the transmitting end, the original information bits sequentially go through the steps of forward error-correction (FEC) encoding and decoding, bit interleaving, mapping, injection level adjustment, and mixing, and then the signal is framed, modulated, and finally sent out. Each layer of an LDM signal shares the same orthogonal frequency division multiplexing (OFDM) signal structure, including inverse fast Fourier transform (IFFT) size, cyclic prefix (CP), and pilot frequency structure. The ATSC 3.0 physical layer standard only uses a two-layer LDM signal structure [27]. The specific implementation has been described in detail in [3].

At the receiving end, a typical successive interference cancellation (SIC) detector is exploited to demodulate the LDM signal, as shown in Figure 1b. First, the most robust CL signal is demodulated, and then, the bit-decoded CL data are subjected to bit-interleaved coded modulation (BICM) (CL regeneration) again. The CL-reproduced signal is subtracted from the delayed received signal in the buffer area to obtain the EL signal and demodulate it. It is easy to see that in order to avoid the spread of errors between layers, the premise of this processing method is that the error rate of CL reception is extremely low, that is, the robustness of the CL signal is required to be very good.

## 3. Proposed En-LDM Scheme

### 3.1. System Model

Under the two-layer LDM signal scenario, due to the fact that the antenna gain and installation height are not always able to best match the conditions of business planning, a large range of EL holes may still occur within the effective coverage range of CL, resulting in inconsistent coverage ranges of different services. Therefore, it is of importance to improve the reception performance of the EL fixed service while maintaining the reception performance of the original CL mobile service. To achieve that goal, the En-LDM scheme in the following is proposed by multiplexing part of the data of the fixed service stream in the upper layer of the LDM. 

**Figure 2:**
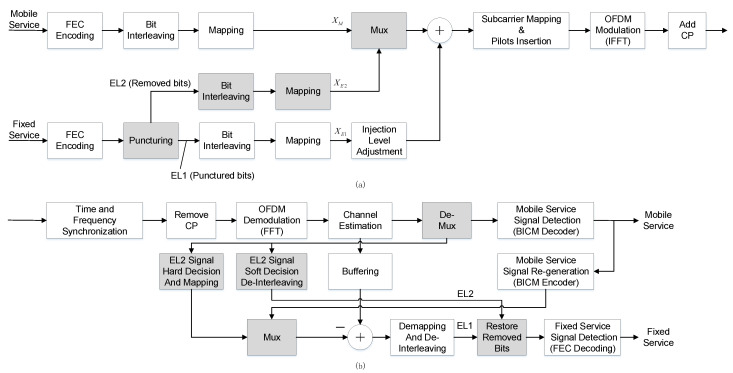
Block diagram of the enhanced LDM (En-LDM) scheme transceiver: (**a**) transmitter: En-LDM scheme that punctures LDPC coded bits before symbol modulation; (**b**) receiver.

Figure 2 depicts the block diagram of the En-LDM transceiver. In Figure 2, in addition to the modules defined in the traditional LDM scheme, the additional modules in whole colored shadow are added to deal with fixed services. In the En-LDM scheme, the CL of the LDM signal transmits both mobile services and fixed services, while the EL only bears fixed services. The fixed service transmitted by CL consists of punching bits, while the fixed service carried by EL is composed of the bits left after punching. Specifically, according to the puncturing method, the data used for the fixed service flow are divided into two parts: EL1 and EL2. EL1 is assigned to the lower layer of the LDM, while EL2 is used for mobile traffic, whose data symbols are sent on top of the LDM signal in an FDM manner. In this case, the SNR of EL2 is much higher than that of EL1, which will facilitate the correctness of decoding.

#### 3.1.1. En-LDM Transmitter

The block diagram of the transmitter of the proposed En-LDM scheme is shown in Figure 2a. Each service can flexibly choose the appropriate FEC coding, BICM scheme according to the requirements, and the selection of the used BICM scheme for the puncturing part of the data can also be agile. The data stream of mobile service uses a BICM encoder to generate quadrature phase-shift keying (QPSK) or non-uniform quadrature amplitude modulation (NU-QAM) symbols, $X_{M}(k)$. The fixed service data stream can be divided into two parts through bit puncturing after FEC encoding; i.e., the punctured bits (Punctured bits) are called EL1, and the removed bits (Removed bits) are called EL2. The symbols of EL1 and EL2 after bit interleaving and mapping are $X_{E1}(k)$ and $X_{E2}(k)$, respectively. The symbols $X_{C}(k)$ generated by the multiplexing of $X_{M}(k)$ and $X_{E2}(k)$ are allocated to CL of LDM in the frequency domain. The symbols $X_{C}(k)$ of CL can be expressed as follows:

(1) $$X_{C}(k)=\{\begin{array}{l} X_{M}(k),k\leq L-1\\ X_{E2}(k-L),k>L-1 \end{array}$$

where k represents the subcarrier index, and L denotes the number of subcarriers occupied by the mobile service symbol $X_{M}(k)$ in the LDM-CL. 

While $X_{E1}(k)$ is assigned to LDM’s EL, the transmitted superimposed symbol $X_{LDM}(k)$ modulated by LDM is expressed as follows:

(2) $$X_{LDM}(k)=\sqrt{\frac{1}{1+\alpha }}X_{C}(k)+\sqrt{\frac{\alpha }{1+\alpha }}X_{E1}(k)$$

(3) $$\Delta =10\cdot \log_{10}(\frac{1}{\alpha })$$

where k represents the subcarrier index, α expresses the LDM average power ratio, and Δ stands for the power injection level, respectively. $X_{C}(k)$ and $X_{E1}(k)$ respectively represent the transmission symbols used for CL and EL on the k-th subcarrier, with the average powers of $X_{C}(k)$ and $X_{E1}(k)$ both normalized. 

After the LDM symbols are generated, subcarrier mapping and pilot insertion are performed to generate an OFDM frame, which is then transformed to the time domain through an inverse fast Fourier transform (IFFT) module with a cyclic prefix (CP) added. CP can deal with inter-symbol interference (ISI) as a result of the multipath fading.

#### 3.1.2. En-LDM Receiver

The block diagram of the receiver in the proposed En-LDM scheme is shown in Figure 2b. After the signal is synchronized in time and frequency, CP removal, fast Fourier transform (FFT) de-mapping, channel estimation, and equalization, etc., the CL signal of LDM is first demodulated. At the receiver, the received signal is expressed as

(4) $$Y_{LDM}(k)=\sqrt{\frac{1}{1+\alpha }}X_{C}(k)\cdot H(k)+\sqrt{\frac{\alpha }{1+\alpha }}X_{E1}(k)\cdot H(k)+N(k)$$

where H(k) represents the transfer function, and N(k) stands for the Gaussian noise and other additive interference signals. After the receiver performs channel estimation on the signal, the CL symbol can be demodulated by the following equalization method, i.e.,

(5) $${\hat{X}}_{C}(k)=\frac{Y_{LDM}(k)\sqrt{1+\alpha }}{\hat{H}(k)}=X_{C}(k)\frac{H(k)}{\hat{H}(k)}+\frac{\sqrt{\alpha }X_{E1}(k)\cdot H(k)+\sqrt{1+\alpha }N(k)}{\hat{H}(k)}$$

where $\hat{H}(k)$ indicates the estimated value of the channel frequency response of the k-th subcarrier. Since CL includes mobile service and fixed service, after de-multiplexing the CL demodulation symbol ${\hat{X}}_{C}(k)$ in the frequency domain, the demodulated mobile service symbol ${\hat{X}}_{M}(k)$ and the fixed service symbol ${\hat{X}}_{E2}(k)$ can be obtained.

The mobile service signal is demodulated by the BICM decoder to obtain mobile service information bits. The fixed service signal uses hard decision demodulation to obtain bit information and remaps to obtain the symbol ${\hat{X}}_{E2}(k)$. The decoded mobile service information bits are subjected to BICM encoding to reconstruct the mobile service symbol ${\tilde{X}}_{M}(k)$, which is multiplexed with ${\hat{X}}_{E2}(k)$ and reconstructed to obtain the replica symbol ${\tilde{X}}_{C}(k)$ of CL. The symbol ${\tilde{X}}_{C}(k)$ regenerated by LDM-CL can be expressed as

(6) $${\tilde{X}}_{C}(k)=\{\begin{array}{l} {\tilde{X}}_{M}(k),k\leq L-1\\ {\tilde{X}}_{E2}(k-L),k>L-1 \end{array}$$

where L represents the number of subcarriers occupied by the mobile service symbol ${\hat{X}}_{M}(k)$ in the LDM-CL.

Before generating the copy symbols of the core layer, the LDM symbols are buffered in “Buffering”. The fixed service symbol can be obtained by subtracting the generated duplicate symbol from the buffered LDM symbol. Then, the demodulation of the LDM-EL fixed service symbols can be expressed as follows:

(7) $$\begin{array}{c} {\hat{X}}_{E1}(k)=\frac{Y_{LDM}(k)\sqrt{1+\alpha }}{\hat{H}(k)\sqrt{\alpha }}-\frac{1}{\sqrt{\alpha }}{\tilde{X}}_{C}(k)\\ \;\;\;\;\;\;=X_{E1}(k)\cdot \frac{H(k)}{\hat{H}(k)}+\frac{\Delta_{C}(k)+\frac{\sqrt{1+\alpha }}{\hat{H}(k)}N(k)}{\sqrt{\alpha }} \end{array}$$

(8) $$\Delta_{C}(k)=X_{C}(k)\frac{H(k)}{\hat{H}(k)}-{\tilde{X}}_{C}(k)$$

where $\Delta_{C}(k)$ represents the CL signal left after interference cancellation.

The fixed service symbols ${\hat{X}}_{E1}(k)$ and ${\hat{X}}_{E2}(k)$ are respectively de-mapped and de-interleaved, and then, the fixed service bit information before puncturing is recovered and decoded to obtain the fixed service bit data according to the transmitter bit puncturing rule.

### 3.2. Channel Capacity Analysis

#### 3.2.1. Analysis for LDM

The maximum achievable data rate under a communication link (in bits/s/Hz) is also known as the Shannon limit. The Shannon limit for the additive white Gaussian noise (AWGN) channel is given in [28]:

(9) $$C_{S}=\log_{2}(1+\frac{S}{N})$$

where S and N are the signal average transmit power and noise power respectively.

Assuming that the total transmission power of the En-LDM system is normalized (that is, S=1) and the injection level is Δ dB, then the transmission power of the two layers can be calculated as

(10) $$\begin{array}{l} P_{U}=\frac{10^{\Delta /10}}{1+10^{\Delta /10}}\\ P_{L}=\frac{1}{1+10^{\Delta /10}} \end{array}$$

The CL signal power is regarded as the transmission power of the desired signal, and the EL signal power is regarded as the noise power when calculating the CL channel capacity. Since the working points of CL service and EL service are obviously different, the CL signal can be regarded as error-free elimination when calculating the EL channel capacity.

Following Equation (9), the achievable capacities for the two transmission layers are calculated as follows:

(11) $$\begin{array}{l} C_{U}=\log_{2}(1+\frac{P_{U}}{P_{L}+N})\\ C_{L}=\log_{2}(1+\frac{P_{L}}{N}) \end{array}$$

Therefore, by substituting Equation (10) into Equation (11), the Shannon limit for the two services of the traditional LDM system are calculated as

(12) $$\begin{array}{l} C_{M}^{LDM}=\log_{2}\left(1+\frac{10^{\Delta /10}}{1+N\cdot (1+10^{\Delta /10})}\right)\\ C_{F}^{LDM}=\log_{2}\left(1+\frac{1}{N\cdot (1+10^{\Delta /10})}\right) \end{array}$$

#### 3.2.2. Analysis for En-LDM

In the En-LDM system, mobile services and fixed services removed from puncturing are combined based on frequency division multiplexing and transmitted through the CL of LDM, where the capacity allocation of each service is linearly proportional to the spectrum allocation [29]. The EL of LDM only transmits the remaining fixed service of puncturing. Therefore, the channel capacity for the transmission of mobile services and fixed services can be calculated as

(13) $$\begin{array}{l} C_{M}^{En-LDM}=C_{U}\cdot \frac{B_{m}}{B_{t}}\\ C_{F}^{En-LDM}=C_{L}+C_{U}\cdot \frac{B_{f}}{B_{t}} \end{array}$$

where $B_{t}$ is the bandwidth occupied by a frame of signal, $B_{m}$ and $B_{f}$ are the bandwidth of CL to transmit mobile services and fixed services removed by puncturing, respectively, and $B_{t}=B_{m}+B_{f}$.

Therefore, by substituting Equations (10) and (11) into Equation (13), the Shannon limit for the two services of the En-LDM system are calculated as

(14) $$\begin{array}{l} C_{M}^{En-LDM}=\log_{2}\left(1+\frac{10^{\Delta /10}}{1+N\cdot (1+10^{\Delta /10})}\right)\cdot \frac{B_{m}}{B_{t}}\\ C_{F}^{En-LDM}=\log_{2}\left(1+\frac{1}{N\cdot (1+10^{\Delta /10})}\right)+\log_{2}\left(1+\frac{10^{\Delta /10}}{1+N\cdot (1+10^{\Delta /10})}\right)\cdot \frac{B_{f}}{B_{t}} \end{array}$$

Equation (14) shows that the distribution of each service capacity in the En-LDM system is only controlled by the signal injection level and the spectrum allocation ratio. Since the power distribution calculation involves the logarithmic calculation of Equation (11), the capacity is allocated for the mobile service and fixed service in a non-linear manner.

#### 3.2.3. BICM Capacity Analysis

Equation (9) gives the Shannon limit, but it does not directly specify the type of signal that is close to the limit. BICM can be used to evaluate the system performance in a binary communication system, which is an effective solution approaching Shannon’s limit with affordable complexity. The BICM capacity can be given by [13]:

(15) $$C_{B,\chi }=m-\sum_{i=1}^{m}E_{b,y}\left[\log_{2}\frac{\sum_{x\in \chi }p(y|x)}{\sum_{x\in \chi_{b}^{i}}p(y|x)}\right]$$

where m is the order of the constellation, χ represents the alphabet of size $M=2^{m}$,$\chi_{b}^{i}$ represents a subset of all signals x∈χ, and its tag has a value b∈{0,1} at the i-th bit. p(y|x) is the transition probability density function (p.d.f.) of the transmitted signal x and the received signal y. In the AWGN channel, p(y|x) is given by:

(16) $$p(y|x)=\frac{1}{\sqrt{2\pi }\sigma }e^{-\frac{{\left(y-x\right)}^{2}}{2\sigma^{2}}}$$

The transmitted symbols need to be normalized, and the following constraints should be met:

(17) $$P_{tx}=\frac{1}{M}\sum {}_{i=1}^{M}{\left|x_{i}\right|}^{2}=1$$

On the premise of satisfying Equations (16) and (17), the BICM capacity obtained by Equation (15) is only determined by the symbol alphabet and the SNR of the signal. The signal power of each service in the En-LDM system obtained by Equation (10) can calculate the SNR of the signal, so the BICM capacity of each service of En-LDM can be calculated. 

## 4. Compute and Simulation Results

### 4.1. Spectral Efficiency Calculation

Following Equations (12) and (15), the spectral efficiency shortfall from Shannon for the mobile service of the traditional LDM and En-LDM systems are calculated as

(18) $$\begin{array}{l} S_{M}^{LDM}=\left(C_{M}^{LDM}-C_{B,\chi 1}\right)/B_{t}\\ \;\;\;=\log_{2}\left(1+\frac{10^{\Delta /10}}{1+N\cdot (1+10^{\Delta /10})}\right)\cdot \frac{1}{B_{t}}-\left(m-\sum_{i=1}^{m}E_{b,y}\left[\log_{2}\frac{\sum_{x\in \chi 1}p(y|x)}{\sum_{x\in \chi 1_{b}^{i}}p(y|x)}\right]\right)\cdot \frac{1}{B_{t}} \end{array}$$

(19) $$\begin{array}{l} S_{M}^{En-LDM}=\left(C_{M}^{En-LDM}-C_{B,\chi 1}\cdot \frac{B_{m}}{B_{t}}\right)/B_{m}\\ \;\;\;\;\;=\log_{2}\left(1+\frac{10^{\Delta /10}}{1+N\cdot (1+10^{\Delta /10})}\right)\cdot \frac{1}{B_{t}}-\left(m-\sum_{i=1}^{m}E_{b,y}\left[\log_{2}\frac{\sum_{x\in \chi 1}p(y|x)}{\sum_{x\in \chi 1_{b}^{i}}p(y|x)}\right]\right)\cdot \frac{1}{B_{t}} \end{array}$$

Obviously, it can be observed from Equations (18) and (19) that the spectral efficiency shortfall from Shannon for the mobile service of the traditional LDM system is equal to that of the proposed En-LDM system. Since the mobile service of the traditional LDM system occupies a higher bandwidth than the En-LDM system, the transmission capacity of the mobile service of the LDM system will be slightly higher.

**Figure 3:**
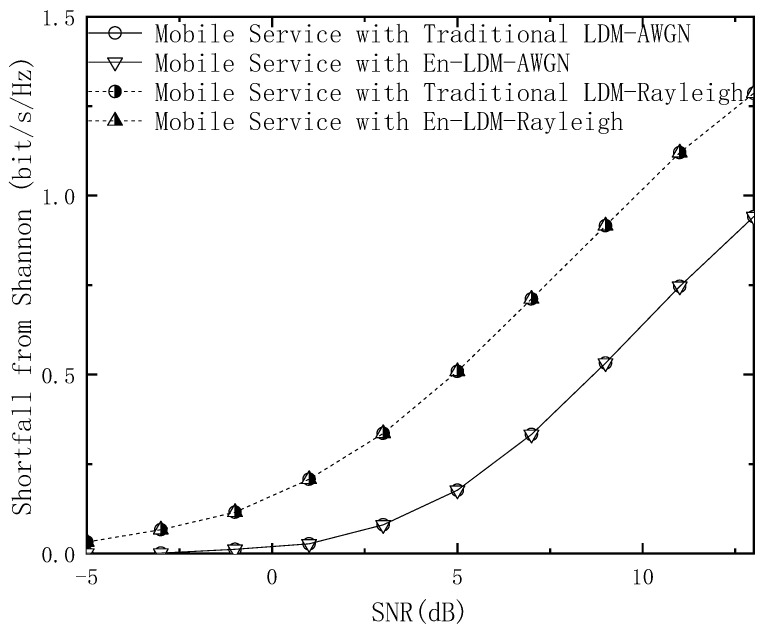
The shortfall from Shannon for the mobile service of the LDM and En-LDM systems under the AWGN and Rayleigh channels, where the parameters of the LDM and En-LDM systems were set to Δ=10 dB, $B_{f}/B_{t}=1/4$, the constellation for the mobile service was set to QPSK.

Figure 3 shows the shortfall from Shannon for the mobile service in the proposed En-LDM and the traditional LDM systems under the AWGN and multipath channels. Note that the system bandwidth is normalized (that is, $B_{t}=1$). The results show that within the working SNR range of the mobile service, the shortfall from Shannon for the mobile service in the En-LDM system is the same as that of the traditional LDM system under the AWGN or Rayleigh channel. In addition, from Equations (18) and (19), it can be seen that the Shannon limit of the En-LDM system and the traditional LDM system under the same bandwidth is the same. Therefore, the spectral efficiency of the mobile service in the En-LDM system is consistent with that of the traditional LDM system. When the SNR is equal to 5 dB, the shortfall from Shannon for the mobile service of the En-LDM/LDM system is 0.51 bit/s/Hz under the Rayleigh channel, while the shortfall from Shannon for the mobile service of the En-LDM/LDM system is 0.18 bit/s/Hz under the AWGN channel.

Following Equations (14) and (15), the spectral efficiency shortfall from Shannon for the fixed service of the traditional LDM and En-LDM systems are calculated as

(20) $$\begin{array}{l} S_{F}^{LDM}=\left(C_{F}^{LDM}-C_{B,\chi 2}\right)/B_{t}\\ \;\;\;=\log_{2}\left(1+\frac{1}{N\cdot (1+10^{\Delta /10})}\right)\cdot \frac{1}{B_{t}}-\left(m-\sum_{i=1}^{m}E_{b,y}\left[\log_{2}\frac{\sum_{x\in \chi 2}p(y|x)}{\sum_{x\in \chi 2_{b}^{i}}p(y|x)}\right]\right)\cdot \frac{1}{B_{t}} \end{array}$$

(21) $$\begin{array}{l} S_{F}^{En-LDM}=\left(C_{F}^{En-LDM}-C_{B,\chi 2}\cdot \left(1+\frac{B_{m}}{B_{t}}\right)\right)/\left(B_{t}+B_{f}\right)\\ \;\;\;\;\;=\log_{2}\left(1+\frac{1}{N\cdot (1+10^{\Delta /10})}\right)\cdot \frac{1}{B_{t}+B_{f}}+\log_{2}\left(1+\frac{10^{\Delta /10}}{1+N\cdot (1+10^{\Delta /10})}\right)\cdot \frac{B_{f}}{B_{t}\left(B_{t}+B_{f}\right)}\\ \;\;\;\;\;-\left(m-\sum_{i=1}^{m}E_{b,y}\left[\log_{2}\frac{\sum_{x\in \chi 2}p(y|x)}{\sum_{x\in \chi 2_{b}^{i}}p(y|x)}\right]\right)\cdot \frac{B_{t}+B_{m}}{B_{t}\left(B_{t}+B_{f}\right)} \end{array}$$

**Figure 4:**
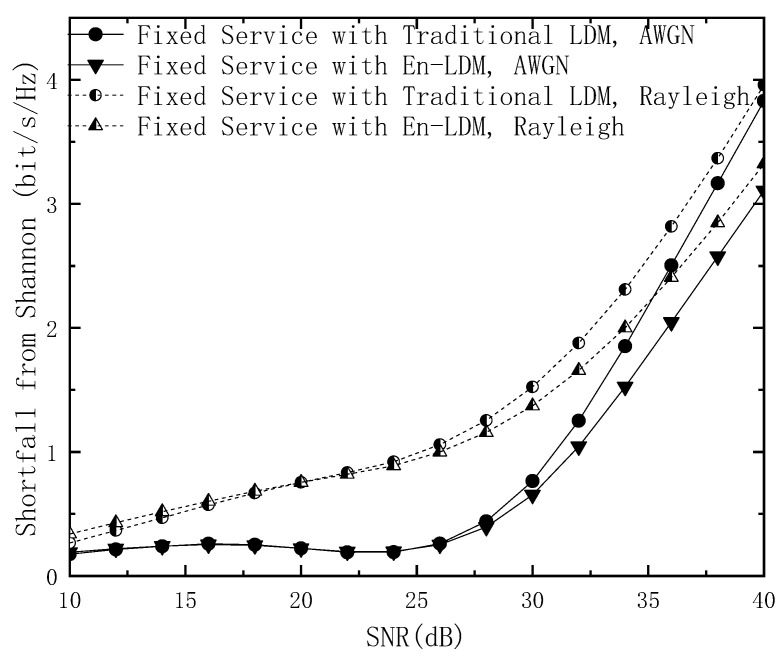
The shortfall from Shannon for the fixed service of the LDM and En-LDM systems under the AWGN and Rayleigh channels, where the parameters of the LDM and En-LDM systems were set to Δ=10 dB, $B_{f}/B_{t}=1/4$, the CL constellation was set to QPSK, and the EL constellation was set to NU−64QAM.

**Figure 5:**
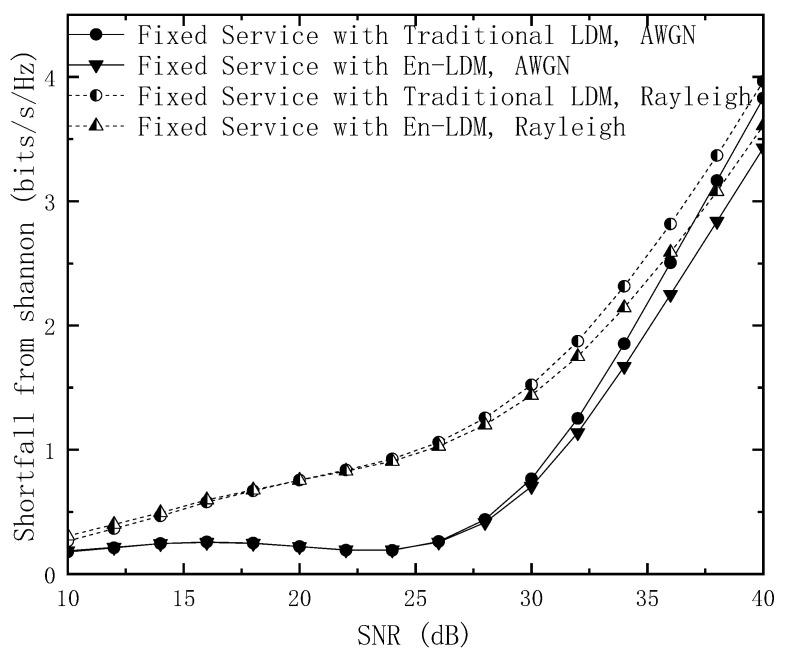
The shortfall from Shannon for the fixed service of the LDM and En-LDM systems under the AWGN and Rayleigh channels, where the parameters of the LDM and En-LDM systems were set to Δ=10 dB, $B_{f}/B_{t}=1/8$, the CL constellation was set to QPSK, and the EL constellation was set to NU−64QAM.

The shortfall from Shannon for the fixed service in the proposed En-LDM and the traditional LDM systems under the AWGN and multipath channels is shown in Figure 4 and Figure 5. It should be noted that the digital modulation constellation used by the fixed service in the En-LDM system is QPSK and NU−64QAM. In Figure 4, the modulation constellation used by the bits extracted by LDPC puncturing is QPSK, which occupies 1/4 of the spectrum resources of the CL. The modulation constellation of the remaining bits of LDPC puncturing is NU−64QAM, and the modulation constellation that carries fixed services in the traditional LDM system is only NU−64QAM. The results show that within the working SNR range of the fixed service, the shortfall from Shannon for the fixed service in the En-LDM system is lower than that of the traditional LDM system under the AWGN or Rayleigh channel. In other words, the En-LDM system has better spectrum efficiency than the traditional LDM system. Specifically, the spectral efficiency for the mobile service with En-LDM system is 0.20/0.22 bit/s/Hz higher than the traditional LDM system under the AWGN/Rayleigh channels when the SNR is 32 dB.

The above conclusion can also be obtained in Figure 5. The modulation constellation used by the bits extracted by LDPC puncturing is QPSK, which accounts for 1/8 of the CL spectrum resources. Another observation can be extracted from Figure 4 and Figure 5; increasing the occupied bandwidth ratio helps the fixed service obtain better spectrum efficiency, which is entirely due to the fact that each layer of services occupies the same time-frequency resources and the increase in the number of LDPC-based punctures can improve the performance of the fixed service.

### 4.2. Simulation Results

In this subsection, the transmission performance of each service of the proposed En-FDM scheme is evaluated under the AWGN and multipath channels. We perform simulation by MATLAB 2017b on the Intel(R) Xeon(R) E5-2667 CPU under a 2.9 GHz and 64-bit Windows 10 operating system. The simulation parameters used in this section are shown in Table 2, and the multipath channel model parameters are shown in Table 3.

**Figure 6:**
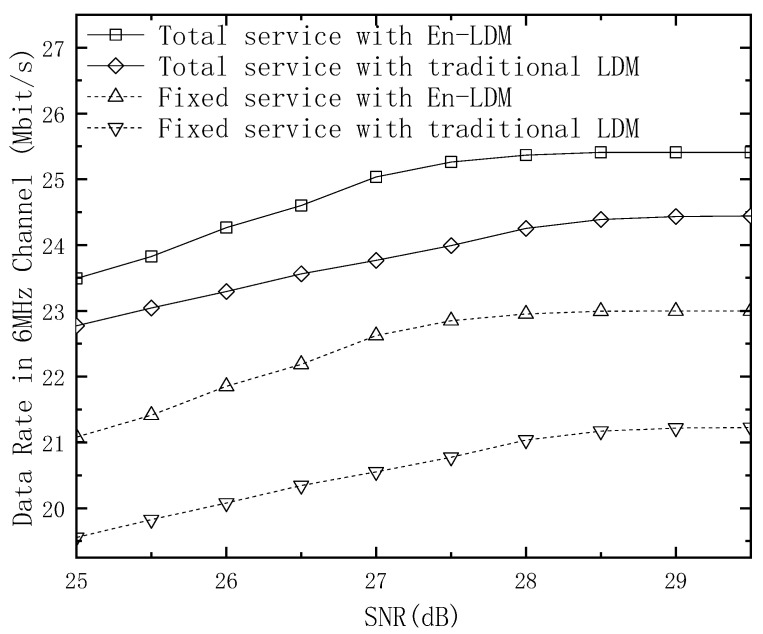
Channel capacity of fixed service and total service of En-LDM and traditional LDM systems under the multipath channel, where the injection level was set to Δ=10 dB, the CL constellation was set to QPSK, the EL constellation was set to NU−64QAM, the puncturing rate of En-LDM scheme was set to 1/13, and the low-density parity-check (LDPC) code rates of CL and EL were set to 5/15 and 11/15, respectively.

The throughput of the mobile service and fixed service of the proposed En-LDM system and traditional LDM system were simulated under the multipath channel. As shown in Figure 6, the En-LDM system provides higher channel capacity for both the overall service and the fixed service (working at high SNR) under the multipath channel. When the SNR is higher than 29 dB, the channel capacity of the fixed service in the En-LDM system is 1.77 Mbit/s higher than that of the traditional LDM system, and the channel capacity of the overall service in the En-LDM system is 0.97 Mbit/s higher than that of the traditional LDM system. In addition, we found that the channel capacity growth of the overall service of the system is less than that of the fixed service, which shows that the channel capacity of the mobile service in the En-LDM system is lower than that of the traditional LDM system. This result is consistent with the previous spectrum efficiency analysis and simulation results. The reason behind this is that the data removed by the puncturing of the fixed service occupies a small number of subcarrier transmissions of the original mobile service in the LDM. As these results show, in a typical scenario of delivering robust HDTV mobile services and high data rate UHDTV fixed services in an radio frequency (RF) channel [30], the proposed En-LDM scheme can provide more efficient spectrum usage while allowing a slight decrease in the transmission capacity for mobile service.

**Figure 7:**
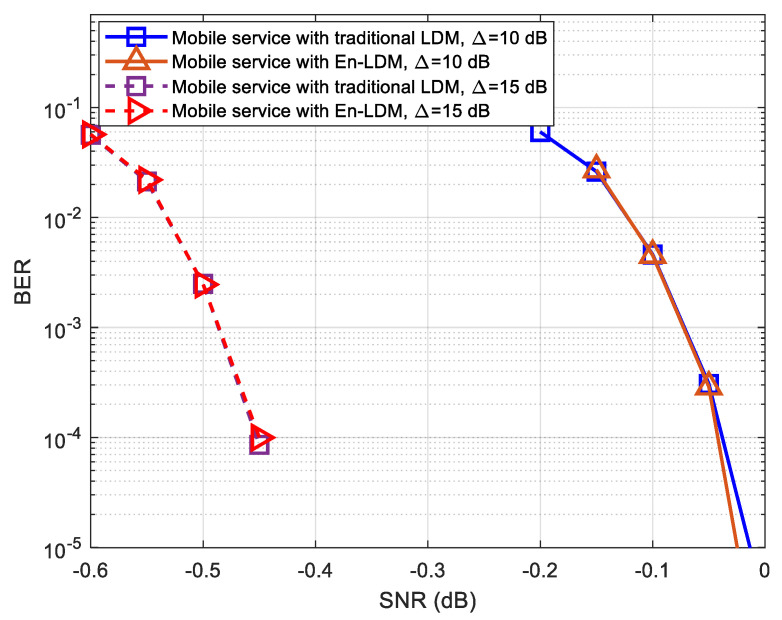
BER performance of mobile service with En-LDM and traditional LDM systems under the additive white Gaussian noise (AWGN) channel, where the injection levels were set to Δ=10 and 15 dB, the core layer (CL) constellation was set to QPSK, the enhanced layer (EL) constellation was set to NU−64QAM, the puncturing rate of the En-LDM scheme was set to 1/13, and the LDPC code rates of CL and EL were set to 5/15 and 11/15, respectively.

**Figure 8:**
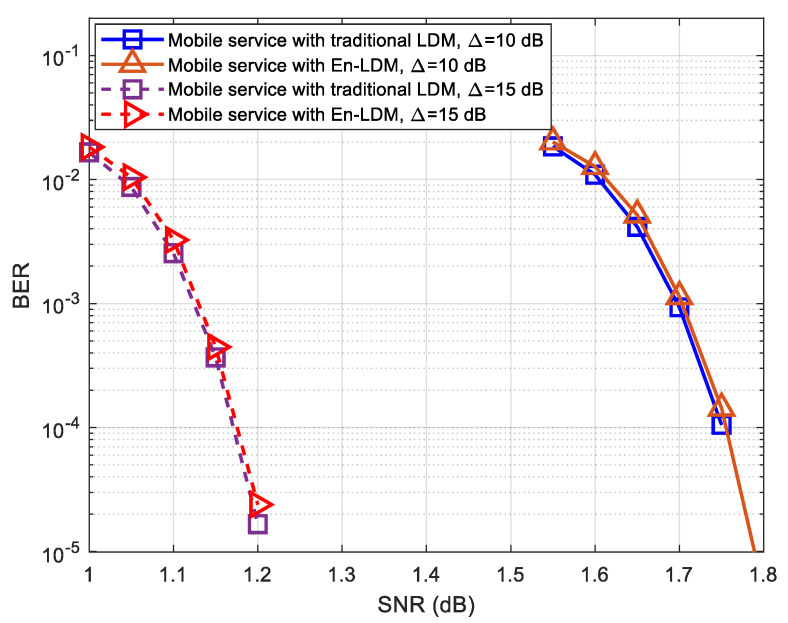
BER performance of mobile service with En-LDM and traditional LDM systems under the multipath channel, where the injection levels were set to Δ=10 dB and 15 dB, the CL constellation was set to QPSK, the EL constellation was set to NU−64QAM, the puncturing rate of the En-LDM scheme was set to 1/13, and the LDPC code rates of CL and EL were set to 5/15 and 11/15, respectively.

**Figure 9:**
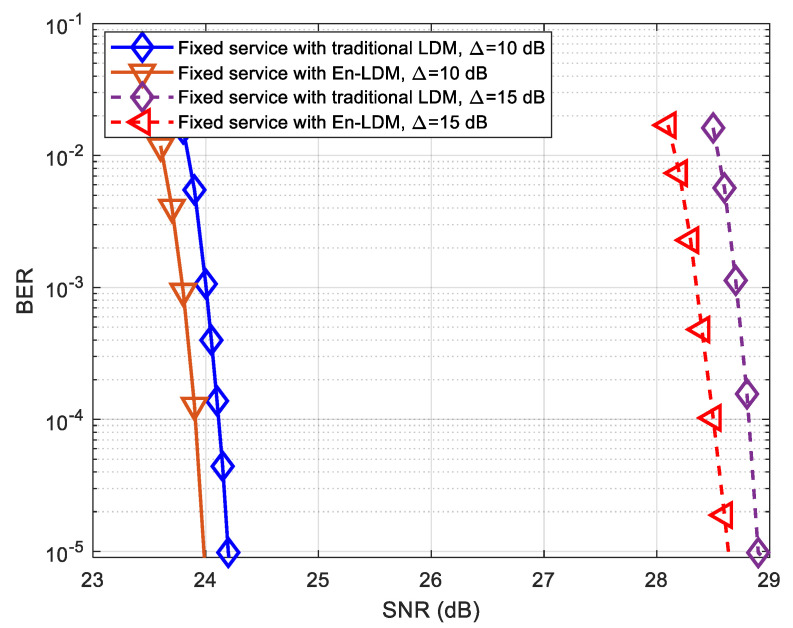
BER performance of fixed service with En-LDM and traditional LDM systems under the AWGN channel, where the injection levels were set to Δ=10 and 15 dB, the CL constellation was set to QPSK, the EL constellation was set to NU−64QAM, the puncturing rate of the En-LDM scheme was set to 1/13, and the LDPC code rates of CL and EL were set to 5/15 and 11/15, respectively.

**Figure 10:**
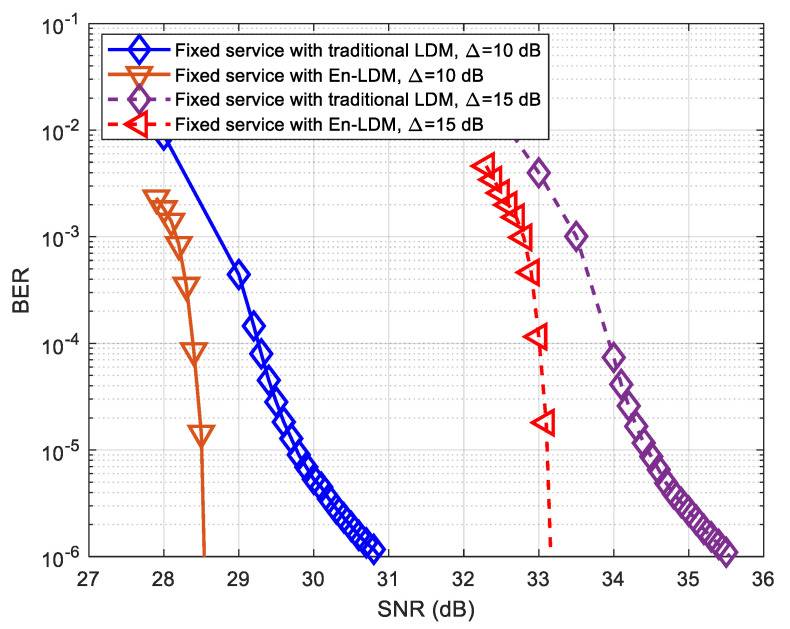
BER performance of fixed service with En-LDM and traditional LDM systems under the multipath channel, where the injection levels were set to Δ=10 and 15 dB, the CL constellation was set to QPSK, the EL constellation was set to NU−64QAM, the puncturing rate of the En-LDM scheme was set to 1/13, and the LDPC code rates of CL and EL were set to 5/15 and 11/15, respectively.

**Figure 11:**
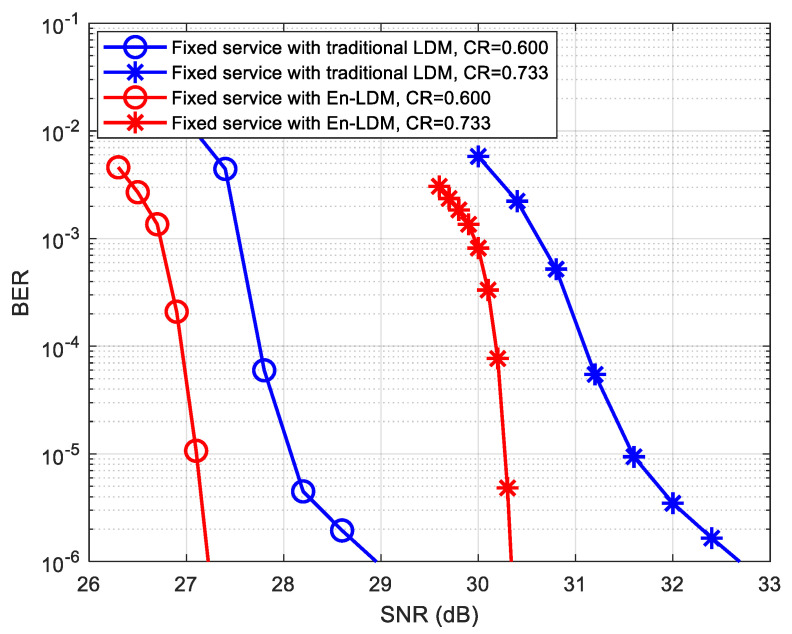
BER performance of fixed service with En-LDM and traditional LDM systems in the multipath channel under different code rates, where the injection level was set to Δ=12 dB, the CL constellation was set to QPSK, the EL constellation was set to NU−64QAM, the puncturing rate of En-LDM scheme was set to 1/13, and the LDPC code rates of EL were set to CR = 9/15 and 11/15.

The bit error rate (BER) simulations of mobile service and fixed service in the En-LDM system and traditional LDM system under the AWGN and multipath channels are performed, and the results are presented in Figure 7, Figure 8, Figure 9, Figure 10 and Figure 11, respectively.

As shown in Figure 7 and Figure 8, the BER performance of mobile service in the proposed En-LDM system and the traditional LDM system are almost the same under the AWGN channel or multipath channel. For delivering robust mobile service, we considered the injection levels of 10 and 15 dB, where the CL has a power level 10 and 15 dB higher than EL. It can be easily seen from Figure 7 and Figure 8 that when the injection level is increased from 10 to 15 dB, the SNR threshold of mobile service will be reduced by about 0.41 and 0.57 dB under the AWGN and multipath channels, respectively.

As shown in Figure 9 and Figure 10, the BER performance of the fixed service in the En-LDM system is better than that of the traditional LDM. When the LDPC coded bit puncturing rate of fixed service data is set to 1/13, the SNR threshold for fixed service in the En-LDM system is 0.21 and 2.15 dB lower than the traditional LDM system under the AWGN and multipath channels, respectively. In addition, it can be observed that when the injection level is increased from 10 to 15 dB, the SNR threshold of fixed service will increase by about 4.65 and 4.70 dB under the AWGN and multipath channels, respectively. This means that in the case of a higher injection level, the impact of the adjustment of the injection level on the SNR threshold of EL is significantly higher than that of CL. 

The most important finding here is that the BER performance of the fixed service in the En-LDM system converges faster than the traditional LDM system under the multipath channel. This advantage of En-LDM is mainly due to the higher robustness of data extracted based on LDPC coded bit puncturing through core layer transmission, which facilitates correct decoding at the receiving end.

To evaluate the performance of fixed service in the En-LDM system, simulation performances are shown in Figure 11 for fixed service in multipath channels under different code rates. It can be observed that when the LDPC code rate of the EL configuration increases from 9/15 to 11/15, the En-LDM system improves the BER performance of the fixed service from 1.7 to 2.2 dB compared to the traditional LDM system. This implies that En-LDM may perform better than traditional LDM system at high code rates for EL configurations.

## 5. Conclusions

This paper proposed a multi-service transmission scheme called En-LDM, which can support the simultaneous transmission of robust HDTV mobile services and high-data-rate UHDTV fixed services. Comparison with the performance of the traditional LDM system demonstrated the significant performance advantage of the En-LDM, especially in the more practical multipath channel environment. Theoretical analysis and computer simulations showed that the receiving SNR threshold of the fixed services in the En-LDM system was lower than that of the traditional LDM system while maintaining the receiving SNR threshold of mobile services. Eventually, the overall channel capacity of the En-LDM system was 0.97 Mbit/s higher than that of the traditional LDM system with a slightly channel capacity loss of mobile service transmission.

To some extent, it is inferred that the proposed En-LDM system can effectively narrow the coverage gap between mobile services and fixed services at the same RF side without sacrificing the coverage performance of mobile services.

## Figures and Tables

**Table 1:** The comparison among several layered division multiplexing (LDM) schemes.

Scheme	Transmitter	Receiver	Achieved Capacity	Complexity
LDM	LDM	SIC	low	low
LTDM [22]	LDM, TDM	SIC	medium	medium
En-LDM	LDM, FDM, Punctured LDPC	SIC	high	high

**Table 2:** Simulation parameters.

Parameter	Value
FFT Size	16k
Scattered Pilot pattern	SP8_2
Cyclic Prefix (CP)	1/16
CL LDPC Code Length	16,200
CL LDPC Code Rate ($CR_{C}$)	5/15
CL Number of Iterations	50
CL Constellation	QPSK
EL LDPC Code Length	16,200
EL LDPC Code Rate ($CR_{E}$)	9/15, 11/15
EL Number of Iterations	50
EL Constellation	NU−64QAM
Puncturing Rate (PR, γ)	1/13
Injection Level (Δ)	10 dB, 12 dB, 15 dB

**Table 3:** Profile of multipath channel model.

Echo	Power (dB)	Time Delay (μs)
1	−3	0
2	0	0.2
3	−2	0.5
4	−6	1.6
5	−8	2.3
6	−10	5.0

## Data Availability

Not applicable.

## References

[1] Digital Video Broadcasting (DVB); Frame Structure Channel Coding and Modulation for a Second Generation Digital Terrestrial Television Broadcasting System (DVB-T2), V1.4.1. July 2015. https://www.etsi.org/deliver/etsi_en/302700_302799/302755/01.04.01_60/en_302755v010401p.pdf.

[2] Error-Correction, Data Framing, Modulation and Emission Methods for Digital Terrestrial Television Broadcasting. June 2015. https://www.itu.int/dms_pubrec/itu-r/rec/bt/R-REC-BT.1306-7-201506-S!!PDF-E.pdf.

[3] Lee J.-Y., Park S.-I., Kwon S., Lim B.-M., Ahn S., Hur N., Kim H.M., Kim J. (2019). Layered Division Multiplexing for ATSC 3.0: Implementation and Memory Use Aspects. IEEE Trans. Broadcast..

[4] Zhang L., Li W., Wu Y., Salehian K., LaFleche S., Hong Z., Park S.-I., Kim H.M., Lee J.-Y., Hur N. (2018). Using Layered-Division-Multiplexing to Deliver Multi-Layer Mobile Services in ATSC 3.0. IEEE Trans. Broadcast..

[5] Lee J., Park S.I., Kwon S., Lim B., Ahn S., Hur N., Kim H.M., Jeon S., Gomcz-Barquero D. Field Testing of LDM and SHVC Broadcast in ATSC 3.0. Proceedings of the 2018 IEEE International Symposium on Broadband Multimedia Systems and Broadcasting (BMSB).

[6] Regueiro C., Barrueco J., Montalban J., Eizmendi I., Velez M. Improving LDPC decoding performance for ATSC 3.0 LDM profiles. Proceedings of the 2017 IEEE International Symposium on Broadband Multimedia Systems and Broadcasting (BMSB).

[7] Park S., Lee J., Kwon S., Lim B., Ahn S., Kim H.M., Jeon S., Lee J., Simon M., Aitken M. ATSC 3.0 Physical Layer Modulation and Coding Performance Analysis. Proceedings of the 2018 IEEE International Symposium on Broadband Multimedia Systems and Broadcasting (BMSB).

[8] Garro E., Gimenez J.J., Gomez-Barquero D., Park S. Pilot optimization for mobile services in ATSC 3.0. Proceedings of the 2016 IEEE International Symposium on Broadband Multimedia Systems and Broadcasting (BMSB).

[9] Park S., Lim B., Kim Y., Kim H.M., Lee S.H., Choi W., Lee D., Lee S.K., Shin Y.W. ATSC 3.0 LDM-based mobile performance under HPHT metropolitan environment. Proceedings of the 2016 IEEE International Symposium on Broadband Multimedia Systems and Broadcasting (BMSB).

[10] Park S.I., Lee J.-Y., Myoung S., Zhang L., Wu Y., Montalban J., Kwon S., Lim B.-M., Angueira P., Kim H.M. (2016). Low Complexity Layered Division Multiplexing for ATSC 3.0. IEEE Trans. Broadcast..

[11] Zhang L., Li W., Wu Y., Salehian K., Angueira P., Montalban J., Kim H.M., Park S., Lee J., Wang X. Two-layer mobile service performance in LDM-based ATSC 3.0 system. Proceedings of the 2016 IEEE International Symposium on Broadband Multimedia Systems and Broadcasting (BMSB).

[12] Cho S., Hwang Y., Myung S., Yang K. (2016). Low-Complexity Decoding Algorithms for the LDM Core Layer at Fixed Receivers in ATSC 3.0. IEEE Trans. Broadcast..

[13] Kwon S., Park S.I., Lee J., Lim B., Kim H.M., Hur N., Kang J. ATSC 3.0 LDM/TDM performance comparison in fixed reception environment. Proceedings of the 2017 IEEE International Symposium on Broadband Multimedia Systems and Broadcasting (BMSB).

[14] Zhang L., Li W., Wu Y., Xue Y., Sousa E., Park S.-I., Lee J.-Y., Hur N., Kim H.-M. (2020). Using Non-Orthogonal Multiplexing in 5G-MBMS to Achieve Broadband-Broadcast Convergence with High Spectral Efficiency. IEEE Trans. Broadcast..

[15] Zhang L., Wu Y., Li W., Park S., Lee J., Hur N., Kim H. Using Layered-Division-Multiplexing to Achieve Enhanced Spectral Efficiency in 5G-MBMS. Proceedings of the 2019 IEEE International Symposium on Broadband Multimedia Systems and Broadcasting (BMSB).

[16] Islam M.S., Patwary M., Tait R., Peytchev E. Layer division multiplexing for 5G DL transmission within ultra-dense heterogeneous networks. Proceedings of the 2020 IEEE 91st Vehicular Technology Conference (VTC2020-Spring).

[17] Zhang L., Wu Y., Li W., Salehian K., LaFleche S., Wang X., Park S.I., Kim H.M., Lee J.-Y., Hur N. (2018). Layered-Division Multiplexing: An Enabling Technology for Multicast/Broadcast Service Delivery in 5G. IEEE Commun. Mag..

[18] Kim H.J., Kwon S., Kim H., Bae J., Hur N. Performance Analysis of LDM and TDM systems for Three PLPs in DVB-T2. Proceedings of the 2020 International Conference on Electronics, Information, and Communication (ICEIC).

[19] Ahn S., Park S., Lee J., Kwon S., Liml B., Kim H.M., Hur N., Wu Y., Zhang L., Li W. Performance Evaluation of ATSC 3.0 Mobile Service with LDM/TDM Under TU-6 Channel. Proceedings of the 2018 IEEE International Symposium on Broadband Multimedia Systems and Broadcasting (BMSB).

[20] Park S.-I., Lee J.-Y., Lim B.-M., Kwon S., Seo J.-H., Kim H.M., Hur N., Kim J. (2017). Field Comparison Tests of LDM and TDM in ATSC 3.0. IEEE Trans. Broadcast..

[21] Kwon S., Park S.-I., Lee J.-Y., Lim B.-M., Ahn S., Kang J. (2019). Detection Schemes for ATSC 3.0 Transmitter Identification in Single Frequency Network. IEEE Trans. Broadcast..

[22] Lee J.-Y., Park S.-I., Kwon S., Lim B.-M., Kim H.M., Montalbán J., Angueira P., Zhang L., Li W., Wu Y.-Y. (2016). Multiple Service Configurations Based on Layered Division Multiplexing. IEEE Trans. Broadcast..

[23] Wang H. (2016). Discussion on Layer Division Multiplexing Technology and Application. Adv. Telev. Eng..

[24] Chernock R., Whitaker J.C., Wu Y. (2017). ATSC 3.0—The Next Step in the Evolution of Digital Television. IEEE Trans. Broadcast..

[25] Zhang L., Hong Z., Li W., Wu Y., Salehian K., Gomez-Barquero D., Angueira P., Montalban J., Kim H.M., Park S. Capacity analysis of LDM-based DTV system with flexible MIMO configuration. Proceedings of the 2016 IEEE International Symposium on Broadband Multimedia Systems and Broadcasting (BMSB).

[26] Li W., Wu Y., Laflèche S., Salehian K., Zhang L., Florea A., Park S., Lee J., Kim H., Hur N. Coverage study of ATSC 3.0. Proceedings of the 2017 IEEE International Symposium on Broadband Multimedia Systems and Broadcasting (BMSB).

[27] Park S.I., Montalbán J., Zhang L., Gil U., Wu Y., Angulo I., Salehian K., Rong B., Li W., Kim H.M. Hardware Implementation and Complexity Analysis of Layered Division Multiplexing (LDM) System for ATSC 3.0. Proceedings of the NAB Broadcast Engineering Conference.

[28] Shannon C.E. (1948). A Mathematical Theory of Communication. Bell Syst. Tech. J..

[29] Zhang L., Wu Y., Li W., Kim H.M., Park S., Angueira P., Montalban J., Velez M. Channel capacity distribution of Layer-Division-Multiplexing system for next generation digital broadcasting transmission. Proceedings of the 2014 IEEE International Symposium on Broadband Multimedia Systems and Broadcasting.

[30] Regueiro C., Montalban J., Barrueco J., Velez M., Angueira P., Wu Y., Zhang L., Park S.-I., Lee J.-Y., Kim H.M. (2016). LDM Core Services Performance in ATSC 3.0. IEEE Trans. Broadcast..

